# HitKeeper, a generic software package for hit list management

**DOI:** 10.1186/1751-0473-2-2

**Published:** 2007-03-28

**Authors:** Jörg Hau, Michael Muller, Marco Pagni

**Affiliations:** 1 Nestlé Research Center, Department of BioAnalytical Science, PO Box 44, CH-1000 Lausanne 26, Switzerland; 2 EPFL Database Laboratory, CH-1015 Lausanne, Switzerland; 3 Swiss Institute of Bioinformatics, Vital-IT group, CH-1015 Lausanne, Switzerland

## Abstract

**Background:**

The automated annotation of biological sequences (protein, DNA) relies on the computation of hits (predicted features) on the sequences using various algorithms. Public databases of biological sequences provide a wealth of biological "knowledge", for example manually validated annotations (features) that are located on the sequences, but mining the sequence annotations and especially the predicted and curated features requires dedicated tools. Due to the heterogeneity and diversity of the biological information, it is difficult to handle redundancy, frequent updates, taxonomic information and "private" data together with computational algorithms in a common workflow.

**Results:**

We present *HitKeeper*, a software package that controls the fully automatic handling of multiple biological databases and of hit list calculations on a large scale. The software implements an asynchronous update system that introduces updates and computes hits as soon as new data become available. A query interface enables the user to search sequences by specifying constraints, such as retrieving sequences that contain specific motifs, or a defined arrangement of motifs ("metamotifs"), or filtering based on the taxonomic classification of a sequence.

**Conclusion:**

The software provides a generic and modular framework to handle the redundancy and incremental updates of biological databases, and an original query language. It is published under the terms and conditions of version 2 of the GNU Public License and available at .

## Background

The automated annotation of protein or DNA sequences is performed using a rather heterogeneous collection of motif predictors, which include regular expressions, generalized profiles, hidden Markov models and neural networks. Since the search for hits by a motif on a sequence is expensive in terms of processing time, the lists of hits obtained by comparing collections of motifs with collections of sequences are usually stored and distributed as dedicated databases. InterPro [1] is a canonical example of such a public resource that, by definition, covers only publicly available sequences and motifs.

However, biological research is often carried out using sequences and motifs that are derived from public, as well as private, sources. There is a clear need to incorporate both sources of data into the same workflow; however, since the software used to generate, manage and keep the public data up-to-date is usually not available, it is difficult to reproduce and maintain similar hit lists locally.

Further issues that complicate matters are the update frequency of public databases, which can lead to an almost continuous data flow, and the redundancy between different databases. As an example, redundancy is visible by the fact that the same protein sequence may appear in different entries, in different databases, or in different releases of the same database. Since most computations are CPU-expensive, repeating the same computation should obviously be avoided.

To satisfy these requirements and to simplify the in-house management of data from very different sources in various formats, we have developed *HitKeeper*. It is a software for the fully automatic handling of multiple sequence and motif databases, as well as classification (taxonomy) information, on a large scale. In addition, *HitKeeper *implements an elaborate and original query syntax to retrieve information. The distribution provides the core programs, a number of test scripts, and a manual with detailed instructions for the set-up of a pilot installation. Since the software architecture is designed to be customizable and extensible, it should be relatively easy for a user with some proficiency in the Perl programming language to introduce new data types and algorithms into the system.

## Implementation

### Program architecture

The following description is focused on the essential features and algorithms of *HitKeeper*. The installation and a number of technical details are explained in-depth in the *Reference Manual*, which is part of the distribution package.

*HitKeeper *is a collection of scripts that interact as concurrent clients with a relational database management system (RDBMS). The software is mostly written in Perl and was developed and tested under various flavours of Linux and Mac OS X using MySQL as RDBMS; it is currently being extended to other RDBMS.

**Figure 1:**
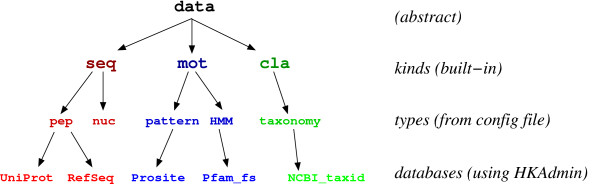
**HitKeeper ontology. **Logical organization of the data and software. See text for discussion.

Fig. 1 is a schematic representation of the logical organization of *HitKeeper*. Starting from the abstract concept of "data", the structure is built around three *kinds *whose properties are reflected in the organization of the software and that are hardcoded in the application: *seq*, biological sequences; *mot*, motifs for predicting hits on the sequences, and *cla*, hierarchical classification (currently limited to taxonomy).

For each *kind*, *HitKeeper *allows multiple *types *of data to be dealt with. As an example, *seq *allows multiple types of sequence data, such as "pep" (for peptide) and "nuc"(for nucleotide). Similar to this, *mot *may comprise the type "pattern" as well as "profile" or "HMM". All types, and all computation algorithms between them (*e.g*. which program is used to run a pattern search on a peptide sequence), are defined in a central configuration file. Besides some general parameters (database server, etc.), this file also holds the list of the modules and external programs that are used (a) for parsing the flat files, (b) performing the actual computations, and (c) dispatching and/or mirroring to any external computing elements. Custom modules written in Perl can be added, either derived from the existing modules or written "from scratch". Parsing of the input data is based on *lazy parsing *that extracts only the relevant information. This minimizes the amount of maintenance that might be induced by format changes in the source data. Custom modules can also be provided for the mirroring of the databases to external computing elements (*e.g*. formating for a BLAST server).

Five distinct clients are available. Three of them, *HKLoader*, *HKUpdater *and *HKPublisher*, are used for RDBMS housekeeping and control of the data flow. They operate concurrently in the background, similar to a system daemon. The two other scripts, *HKReader *and *HKAdmin*, are used to interact with the RDBMS. While the former is solely intended for querying the system, the latter also allows the administration of *HitKeeper*; as an example, the *HitKeeper *administrator defines interactively which database (UniProt, Prosite, etc.) is actually parsed and used for the calculations. Both scripts provide the functionality of a command-line tool, and the interactivity of a "shell-like" environment; they accept input from STDIN and can thus be controlled through other scripts and pipes. This allows automation and enables performing tasks in batch mode, either directly from the command line or by reading commands from a file.

### Data lifecycle and computations

**Figure 2:**
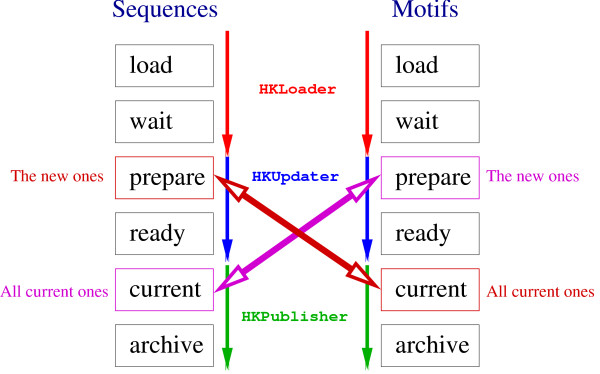
**Schematic representation of the sequence and motif pipelines. **Several successive versions of a given source database usually coexist at different stages in a pipeline. The databases are processed by three scripts running simultaneously, in a manner similar to a system daemon: *HKLoader *watches the source data files for changes (using the date/time stamp). This script is responsible for parsing and converting the raw data, detecting redundancy, and transferring the "clean" data into the SQL database. *HKUpdater *updates the hit list. Once a motif database enters the *prepare *state, the new motifs are computed against the sequences that are in *current *state. Similarly, when a sequence database comes in the states *prepare*, the new sequences are computed against the motifs that are in the *current *state. The two computational tasks, sequences-vs-motifs and motifs-vs-sequences, are never executed simultaneously – this keeps the two pipelines synchronized. Once the calculations are done, *HKPublisher *becomes responsible for the deployment of the databases to external computing elements (*e.g*. a blast server) and the database flagged as *ready *is promoted to *current *("in production"): all subsequent queries are now applied to this database. Previous versions can be kept as archives or deleted to reclaim space.

As mentioned above, *HitKeeper *reads three kinds of input data. Each is associated with a "pipeline" where several versions of a database, such as weekly releases, can coexist. However, only the version with the status 'current' is in the production stage and can be queried. Fig. 2 illustrates how the *seq *and *mot *pipelines are synchronized with respect to the incremental updates of the hit list.

Computations are set up on a per-database basis, so that *all *entries in a given sequence database are expected to be calculated against *all *entries in a motif database. However, not all *databases *are necessarily calculated against each other: the software uses a "subscription" model, defining which database pairs are to be calculated. In this way, it is possible to set up calculations as needed and to adjust the allocation of computing resources.

All hit list computations are performed by calling external software, *i.e*. they are not hardcoded in *HitKeeper*. A simple implementation of a pattern-matching algorithm is provided and can be used as template for custom extensions.

**Figure 3:**
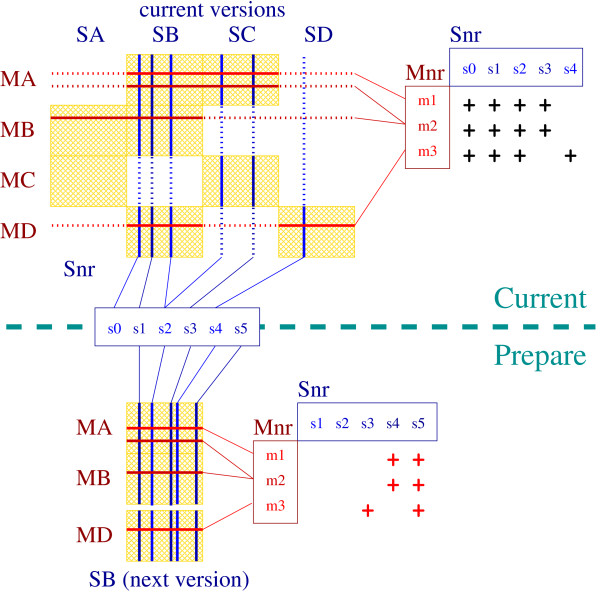
**Example of redundancy management. **This example [9] uses four motif (MA ... MD) and four sequence databases (SA ... SD). The upper part of the figure corresponds to all data that are currently in production. The table on the upper left represents the different databases and some of their individual entries (horizontal and vertical lines). A yellow rectangle symbolizes a subscription for computation. The small table on the right-hand side represents five "non-redundant" sequences ("Snr", arranged in columns) and three motifs ("Mnr", in rows). The computations between individual sequences and motifs are symbolised with black crosses; these do not necessary signal the *presence *of a match, but indicate the fact that the necessary calculations have been performed. - The bottom part of the figure shows a new version of sequence database SB that is being prepared to replace the current version. Sequence s0 will be deleted from database SB, sequence s5 will be inserted, s3 and s4 are new to SB but already present in other databases. The computations that must be performed are indicated by the red crosses. Note that there is no need to compute s4 against m3, since it was already present in SD which, in turn, is already subscribed to MD. - The same principle applies for updating a database of motifs.

If a sequence or motif database is updated, repeating the same computations for sequences or motifs that have not changed should be avoided. This is the purpose of the incremental update algorithm in *HitKeeper*. The algorithm is identical for sequence and motif database updates. Complications arise from the optional subscriptions and from the handling of redundancy; a typical case handled by our algorithm is outlined in Fig. 3.

## Results and discussion

### Installation, validation and scalability

The prerequisites for the installation of *HitKeeper *are the availability of a MySQL server and a few Perl modules from CPAN; according to our experience, the presence of the system administrator is preferable at this stage. The deployment of a *HitKeeper *installation as such is essentially performed through a shell script within a few seconds.

The validation of a *HitKeeper *installation concerns in particular the incremental updates and the query mechanism. Two tests are provided in the distribution and implemented as shell scripts, thus emulating commands as they would be typed by the user instead of querying the RDBMS directly. They should be run as "operational qualification tests" and will verify the correct behaviour of the parser, computation engine, incremental update, and query mechanism.

**Figure 4:**
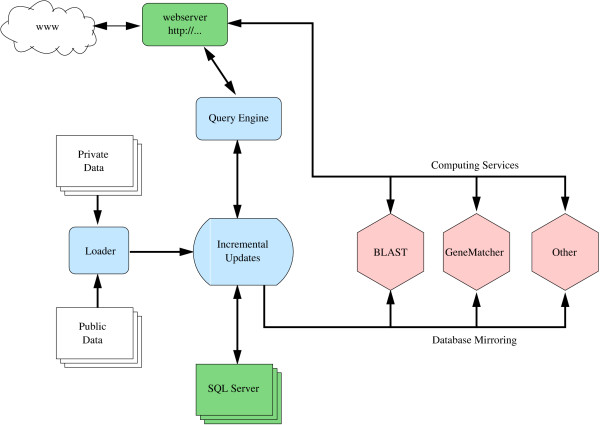
**Schematic structure of the MyHits webserver. **The tasks provided by *HitKeeper *are shown in blue. Services that provide infrastructure (MySQL, Apache) are displayed in green, and computing services in pink. The different tasks are distributed over different hosts, and synchronization of data is controlled by *HitKeeper*.

Historically, *HitKeeper *was developed as the "back end" of the *MyHits *web site [2] as depicted in Fig. 4. *MyHits *has been in production status since 2003 and currently handles more than 7 million non-redundant sequences with weekly updates from a number of major databases, and 21 million hits on these (Table 1). Thus, *HitKeeper *can be considered to be robust and scalable.

**Table 1:** Turnover of sequence data

	db versions	total entries	sequences
current	39	~ 7 · 10^6^	~ 5.7 · 10^6^
total over 9 months	545	~ 122 · 10^6^	~ 8.3 · 10^6^
ratio	0.07	0.06	0.69

Numbers of protein database versions, entries and sequences in *HitKeeper *behind the *MyHits *website, counted at the end of a nine-months period and cumulated over the same time. The ratios indicate frequent updates of the database source files (545 individual releases for a final count of 39 database versions), frequent modifications of the annotations for the complete entries, but a much lower rate of sequence changes (69% remain unchanged over time).

### Queries

*HitKeeper *implements an elaborate and original query syntax to retrieve information. Besides support for logical operators (OR, AND, NOT), *HitKeeper *allows sequences to be retrieved with logical constraints on the arrangement of the motifs found along the sequence. An expression that specifies such a particular "motif of motifs" is called a *metamotif*. Metamotif queries are expressed in a grammar that is specific to *HitKeeper*, yet human readable. This grammar is parsed and then compiled into an SQL query. The metamotif query language was inspired by our experience with *mmsearch *[3] and *twofeat *[4].

While presenting the full query capabilities of *HitKeeper *is out of the scope of this paper, some typical examples of the query language are given below. The setup of the following example dataset is described fully in the Reference Manual; hits can be calculated overnight on a standard Linux workstation. We make use of three common databases: The Swiss-Prot protein sequences (designated with sw hereafter) [5], the Prosite patterns (pat) [6], and the NCBI taxonomy data (taxid) [7,8]. An additional database of "virtual" motifs is automatically derived from the Swiss-Prot "FT" lines (feature) with a script that produces more than 800 of these 'virtual motifs'. The computation yields two 'hit' lists (Swiss-Prot *vs *Prosite, and Swiss-Prot *vs *the ft motifs), and a single 'hat' list (*i.e*. match between sequence and classification): Swiss-Prot *vs *NCBI taxomomy. The latter is used for filtering by taxonomy.

The original text entries can be retrieved using alternative but unique designations. As an example, the Prosite entry for the pattern with id CORNICHON can be retrieved using its name, ID, or accession number:

   mot_fetch_entry pat:CORNICHON

   mot_fetch_entry PS01340

Queries can be "stored" using a query identifier, indicated by -ref=... in the examples below. These identifiers are used to repeat, refine or even string together queries. The following example will refer to all bird sequences from Swiss-Prot:

   hat_query cla_parent=Aves seq_source=sw -ref=$BIRDSEQ

Re-using the same query, count the sequences and the species, then retrieve the non-redundant sequences of all birds in Swiss-Prot:

   query_stat $BIRDSEQ

   seq_fetch_nr $BIRDSEQ

Since the taxonomy data are available, it is easy to list all species covered by that query:

   cla_fetch_desc $BIRDSEQ

A list of all matches of Prosite patterns against these sequences can be obtained as follows:

   hit_query seq_name=$BIRDSEQ mot_source=pat

*HitKeeper *also has the capability to perform negative matching, such as finding all bird sequences with *no *match by any Prosite pattern:

   hit_query seq_source=sw mot_source=pat -ref=$PROSITE

   seq_query seq_name=$BIRDSEQ  not_seq_name=$PROSITE

A simple example is to retrieve all existing hits for the protein VAV_RAT [Swiss-Prot:P54100]:

   hit_query seq_name=sw:VAV_RAT

Note that these include DOMAIN sh2 (one hit), DOMAIN sh3 (two hits) in a particular arrangement. To search all sequences that fulfill a similar arrangement, a metamotif query with the *followed by *operator *~~ *is used:

   mom_query (DOMAIN_sh3~~ DOMAIN_sh2~~ DOMAIN_sh3)

At the time of writing, there are about 22 proteins meeting this criterion in Swiss-Prot.

As another example, Prosite has a pattern pat:THIOREDOXIN that targets the active site of the thioredoxin domain [Prosite:PS00194]. In Swiss-Prot, the thioredoxin domain itself is annotated and was extracted from the FT line as ft:DOMAIN_Thioredoxin. The hit by the pattern is usually present within the domain annotation, but not always. In addition, some domain annotations do *not *include an active site that the pattern would match. The analysis is not straightforward since many proteins have multiple hits with the pattern and domain annotations. The following commands were used to obtain the counts as shown in Table 2:

First, a hit query for hits by either of the two motifs pat:THIOREDOXIN or ft:DOMAIN_Thioredoxin is performed and is saved under the query identifier $all_hit. The comma between the two motif names is the *OR *operator:

**Table 2:** Match counts

#sequences	#motifs	#hits	condition
374	2	737	X OR Y
231	2	542	X /</ Y metamotif
81	1	81	X NOT IN (X /</ Y)
68	1	72	Y NOT IN (X /</ Y)

Counts documenting the occurence of a match by a THIOREDOXIN pattern (X), and by an annotated Thioredoxin domain (Y) in the "FT" lines of Swiss-Prot. The metamotif detects only those matches were X is located within Y. The numbers may change with a different release of Swiss-Prot.

   hit_query -ref=$all_hit  mot_name=pat:THIOREDOXIN,ft:DOMAIN_Thioredoxin

Next a search for hits is carried out where the motif pat:THIOREDOXIN is 'embedded' in the motif ft:DOMAIN_Thioredoxin. Since there are proteins with multiple thioredoxin domains, a metamotif with the *is included *operator /</ was used to associate the pattern and the annotation. As this is a metamotif query, mom query is used instead of hit query:

   mom_query (pat:THIOREDOXIN) /</ (ft:DOMAIN_Thioredoxin)   -ref=$mom_hit

In a third step, hits that contain the motif pat:THIOREDOXIN but that are not included in those that bind the metamotif are identified:

   hit_query mot_name=pat:THIOREDOXIN  not_hit_list=$mom_hit -ref=$pat_not_mom

and the last dataset consists of hits with the motif ft:DOMAIN_Thioredoxin, but that are not in the metamotif hit list:

   hit_query mot_name=ft:DOMAIN_Thioredoxin   not_hit_list=$mom_hit -ref=$ft_not_mom

Finally, the results of all four queries are reported:

   query_stat $all_hit $mom_hit $ft_not_mom  $pat_not_mom

The result is summarised in Table 2 and shows that there are 81 matches by patterns that are not included in the matches by domains. On the other hand, there are 72 domains where the corresponding pattern is not present. The execution time of this last example is typically only a few seconds.

## Conclusion

*HitKeeper *provides a generic, modular and extensible framework to handle the redundancy and incremental updates of biological databases and calculations between them. It allows any user to manage his/her own "private" collections of protein sequences and motifs, in addition to the public ones. *HitKeeper *implements an elaborate query syntax to retrieve information. These queries enable the user to specify constraints for searching proteins, such as retrieving sequences that contain specific motifs, or a defined arrangement of motifs ("metamotifs"), or queries based on the classification of sequences.

While it is not a "ready-to-use" annotation software, the system is designed to be modular, extensible and scalable. New data formats can easily be incorporated by writing custom parsers. The command-line interface of *HitKeeper *allows straightforward integration and interaction with standard tools in the Unix environment, such as scripting, piping, etc.

*HitKeeper *is used at the production stage in the "back-end" of the *MyHits *web site. Hence it is actively maintained; bug fixes and new functionalities are being added into the distribution on a regular basis.

## Availability and requirements

Project name: HitKeeper

Project home page: 

Operating system: Linux, Mac OS X, Solaris

Programming language: Perl, bash, SQL

Other requirements: MySQL 4.1 or higher, a few Perl modules from CPAN

License: GNU General Public License version 2

Any restriction to use by non-academics: None

## Competing interests

The author(s) declare that they have no competing interests.

## Authors' contributions

MP had the original idea and implemented most of the software. MM investigated the incremental update algorithm and the query language [9]. JH developed the setup and testing procedures and wrote the documentation. All authors read and approved the final manuscript.
